# Supplementary material for: Rapid turnover of life-cycle-related genes in the brown algae
Source: Genome Biol. 2019 Feb 14;20:35. doi: 10.1186/s13059-019-1630-6 (PMC6374913; doi:10.1186/s13059-019-1630-6)
Supplement: Supplementary file 3 — Protein alignments, corresponding Gblocks html file and CDS sequences for the lineage conserved gametophyte-biased genes. (ZIP 2109 kb) [file 13059_2019_1630_MOESM3_ESM.zip › Con_GA_alignments/Ec-11_001850.aa_ali.fasta-gb.htm]

Ec-11\_001850.aa.fasta.trans.aa\_ali.fasta


## Gblocks 0.91b Results

Processed file: **Ec-11\_001850.aa.fasta.trans.aa\_ali.fasta**  
Number of sequences: **2**  
Alignment assumed to be: **Protein**  
New number of positions: **357** (selected positions are underlined in blue)

```
                         10        20        30        40        50        60
                 =========+=========+=========+=========+=========+=========+
Ec-11_001850     MGPTTKDIGMILDGAAGGIDEGSGRLHLDMAQEDLEEELRTQPLLDSVRSLVNKERQLAG
MSTRG.19158.1    ------------------------------------------------------------
                                                                             


                         70        80        90       100       110       120
                 =========+=========+=========+=========+=========+=========+
Ec-11_001850     LQLEEKEREAELWRDRFRQLQIESGGIDSAAREAGETIATESWHRDEEDSDWDNLQAASK
MSTRG.19158.1    ------------------------------------------------------------
                                                                             


                        130       140       150       160       170       180
                 =========+=========+=========+=========+=========+=========+
Ec-11_001850     KAGDRYLGKLRRQESLDLSRRGIGKEDLEGIVVKLRWLRGFTSVDLRGNNIDDACVQPLK
MSTRG.19158.1    ------------------------------------------------------------
                                                                             


                        190       200       210       220       230       240
                 =========+=========+=========+=========+=========+=========+
Ec-11_001850     SLMALRQLCSLDLRGNQLGPSSGNALLESLSRCRRLQVLRLEANGLFARTPGAGARLAAG
MSTRG.19158.1    ------------------------------------------------------------
                                                                             


                        250       260       270       280       290       300
                 =========+=========+=========+=========+=========+=========+
Ec-11_001850     LKAAIKAGGCRHLWSLSVTLDDFDGNFFMNAPRKTKAKTAGGGPTATGTGRSTRKAAGPP
MSTRG.19158.1    ------------------------------------------------------------
                                                                             


                        310       320       330       340       350       360
                 =========+=========+=========+=========+=========+=========+
Ec-11_001850     PAAGPMPAAHPMNALALARAFHPRAPDGGAGALLNEDGESEANSRGVRGNTKRGSARVGR
MSTRG.19158.1    ----------PMNALTFTRAFHPRASGGGLGAFSSGGDGAGSGGDGGKGGKGRGESNNN-
                           ######################                            


                        370       380       390       400       410       420
                 =========+=========+=========+=========+=========+=========+
Ec-11_001850     GSITSLGLPYARLHPRTVEELAAHAISSLTHLDLSFCYIGPAGAVALARGLDGHDGGSSR
MSTRG.19158.1    GCITSLGLPYARLHPRTVEELAAYAISSLTHLDLSFCYVGPAGAAALARALDGSEGNNDK
                 ############################################################


                        430       440       450       460       470       480
                 =========+=========+=========+=========+=========+=========+
Ec-11_001850     GSGTHGRGSRSLRSLQLPHNAVGDTGAHALGQSLSSNRCLTFLSLASNGIGPAGARALAA
MSTRG.19158.1    GSTARGRGSRSVRSLKLPHNAVGDSGARAFSRALQSNRCLTSLSLASNGIGPIGGHALAA
                 ############################################################


                        490       500       510       520       530       540
                 =========+=========+=========+=========+=========+=========+
Ec-11_001850     AVGSRGNDGGGSVLTRLDMGDNPLEGEAARLLVAAATRQAPTD-----GHGGKDGDG-TT
MSTRG.19158.1    VVGR-----GSNALARLDVADNPLGGDASRHLVTSATAGAAGDSPASTGNGGSDRKGGPA
                 ###      ##################################                 


                        550       560       570       580       590       600
                 =========+=========+=========+=========+=========+=========+
Ec-11_001850     RGATTLQVLGLDRALGAAVATREAARRAASAAAACEAATTTTLVAIGNEVSVQPEDAIGN
MSTRG.19158.1    CEGVTVRIDGLDRVAGATVAT----MVGAEAGTTAVAHGSNVLLAVDKEVVVQSGD---D
                     #################       ############################    


                        610       620       630       640       650       660
                 =========+=========+=========+=========+=========+=========+
Ec-11_001850     GGLVQVYQVLLDSRFEMPADEIFTIRGGAKGRGATLDIEWSCATDWLDPCWWRVSQTRKG
MSTRG.19158.1    GRLDQVYQIILDSRFELPAHQRSSSGASEMDRESTLDIRWGVTTSWLGACEWRVSRKREG
                 ###################            #############################


                        670       680       690       700       710       720
                 =========+=========+=========+=========+=========+=========+
Ec-11_001850     QPELSVKEGVGTEEGWVSGQANHDGGELRDGYRCQRL-----------------------
MSTRG.19158.1    EPEFSVKEGVGAGKRLAVGLGENDGDEPRDGHSPIRLQSNGWRKHDLLKIFVRPPRGGAG
                 #####################################                       


                        730       740       750       760       770       780
                 =========+=========+=========+=========+=========+=========+
Ec-11_001850     --------------------------------GAPSTQESSTRRLNRPRPIS--------
MSTRG.19158.1    PVSCCDLTIGLMKSEPLPATSMAYGLLWADPEGVDGDVQSKCRWPEEDRPRSPSAAECAE
                                                 ####################        


                        790       800       810       820       830       840
                 =========+=========+=========+=========+=========+=========+
Ec-11_001850     -------------------------------MQRVEKTRHSQGFRPASDWQRRWAREVL-
MSTRG.19158.1    VAFQMSSDTAPFSAASEYSKARYTRINCAGDMRRLRQVQIGHGGRIRASWQAMLVTLQLS
                                                ############################
```

```
Parameters used
Minimum Number Of Sequences For A Conserved Position: 2
Minimum Number Of Sequences For A Flanking Position: 2
Maximum Number Of Contiguous Nonconserved Positions: 8
Minimum Length Of A Block: 10
Allowed Gap Positions: None
Use Similarity Matrices: Yes
```

```
Flank positions of the 9 selected block(s)
Flanks: [311  332]  [361  483]  [490  523]  [545  561]  [569  596]  [601  619]  [632  697]  [753  772]  [812  839]  

New number of positions in Ec-11_001850.aa.fasta.trans.aa_ali.fasta-gb:  357  (42% of the original 840 positions)
```
